# Editorial for Special Issue: Highly Efficient Energy Harvesting Based on Nanomaterials

**DOI:** 10.3390/nano12091572

**Published:** 2022-05-06

**Authors:** Seok Woo Lee

**Affiliations:** School of Electrical and Electronic Engineering, Nanyang Technological University, Singapore 639798, Singapore; sw.lee@ntu.edu.sg

Energy-harvesting systems generate electricity or produce fuels such as hydrogen from various energy sources such as thermal energy, kinetic energy, and renewable energy. For several decades, many researchers have studied the conversion of such energy for use in applications on various scales, from mega-watt grid systems to the micro-watt internet of things (IoTs). This Special Issue entitled “Highly Efficient Energy Harvesting Based on Nanomaterials” presents seven peer-reviewed journal papers written by outstanding authors worldwide [1,2,3,4,5,6,7]. These novel studies cover various topics related to thermal energy management, kinetic energy harvesting, and electrochemical energy conversion.

Phase-change materials (PCMs) have important roles; they compensate for large fluctuations in the temperature of thermal energy-harvesting systems for highly efficient heat conversion. This issue presents three research papers that concern high-performance PCMs and their design to improve uniform temperature distribution for uniform phase change. Al-Najjar et al. [1] demonstrated the effective thermal energy management of photovoltaic (PV) modules using an improved melting duration for phase-change materials (PCMs). As a large amount of solar energy is wasted to heat in PV systems, PCMs have been used for thermal storage, managing the temperature of PV modules for long lifespans. This study reported a new mathematical model for PV/PCM metal foam assembly to improve melting duration. The results of computational fluid dynamics (CFD) agreed with the experimental validations. A noticeable decrease in PV cell temperature with a corresponding improvement in PCM melting duration was observed. Thus, a metal foam layer in PV/PCM systems would be beneficial at low solar radiation, low ambient temperature, or high wind speed. Najim et al. [2] introduced fin arrays to improve the thermal response of a PCM in vertical tripe-tube casing. Sluggish response rates during the phase change of PCMs are caused by high storage density and little temperature degradation. To mitigate this challenge, fast heat exchange is necessary. This study optimized the fin structures, distribution patterns, and spatial zones of a heat exchanger. The results showed that varying the dimension of the fin with the heat flow direction enabled a faster charging rate, higher storage rate, and more uniform temperature distribution than fins with uniform dimensions. Yu et al. [3] employed a graphene aerogel-supported PCM composite for highly efficient thermoelectric energy harvesting. The PCM composites maintained the stable temperature gradient of the thermoelectric power generator under an external temperature change. The graphene nano-platelet filler improved the thermal conductivities of PCM composites. The authors suggested that this system could be used as a thermal sensor, a heat recovery device, and a functional power generator after further optimization.

Kinetic energy harvesting using nanomaterials has been studied for use in various applications for several decades. This issue introduces two research papers concerning a triboelectric energy harvester and a piezoelectric energy harvester using nanomaterials. Gallardo-Vega et al. [4] presented a triboelectric energy harvester (THE) formed with a stainless-steel substrate with a molybdenum disulfide (MoS_2_) film coating for the top element and a polydimethylsiloxane (PDMS) film deposited on an indium tin oxide electrode. It demonstrated kinetic energy harvesting for a mechanical vibration of 59.7 Hz to supply the maximum power of 0.11 mW using a load resistance of 47 kΩ. This harvester is expected to be applied to smart healthcare devices. Li et al. [5] investigated the temperature-dependent longitudinal piezoelectric coefficient of K_0.5_Na_0.5_NbO_3_ single crystals via the Landau–Ginzburg–Devonshire theory. K_0.5_Na_0.5_NbO_3_ is a promising lead-free piezoelectric ceramic that could be used for environmentally friendly power supply to wearable electronics. This study revealed the temperature dependency of the piezoelectric anisotropy of K_0.5_Na_0.5_NbO_3_. This result provides insight into the phase change of piezoelectric materials in terms of temperature and also informs strategies in the optimization of piezoelectric materials and devices.

Electrochemical systems are widely used for not only energy storage but also various energy-harvesting systems such as hydrogen production, thermal energy harvesting, and various fuel cells. This issue presents two research papers concerning a highly efficient catalyst for water splitting and a fabrication method for microelectrochemical devices with ion-hosting electrode materials for the evaluation of their electrochemical characteristics. Rehman et al. [6] proposed a facile strategy to synthesize bifunctional catalysis for both oxygen evolution reaction (OER) and hydrogen evolution reaction (HER). Simply annealing the precursors induced the in situ conversion of the heterojunction along with surface-induced oxygen vacancies and synthesized oxygen-defect-rich Co_9_S_8_/CoO hetero-nanoparticles with a nitrogen-doped carbon shell (ODR-Co_9_S_8_/CoO/NC). This study provided new insight into design of cost-effective, novel, metal-free electrocatalysts. Yun et al. [7] introduced a thin film deposition method for copper hexacyanoferrate (CuHCFe) thin film, a Prussian blue analogue (PBA). PBAs are widely used in electrochemical energy storage and various energy harvesting applications due to their fast kinetics. For further investigation into ion transportation in PBAs, this study fabricated a microelectrochemical device with a CuHCFe thin film electrode using the introduced deposition method. The proposed method was shown to be able deposit a PBA thin film on any surface, including insulating substrate, and its usage could be extended to various applications.

This issue would not have been successful without the authors’ contributions of research articles and review papers. We hope that the readers can gain an insight into the various studies that have been carried out in the field of energy harvesting.
